# Cavernous sinus thrombosis caused by contralateral sphenoid sinusitis: a case report

**DOI:** 10.1186/1746-160X-9-9

**Published:** 2013-03-13

**Authors:** Hiroaki Komatsu, Fumihiko Matsumoto, Misato Kasai, Kaori Kurano, Daisuke Sasaki, Katsuhisa Ikeda

**Affiliations:** 1 Department of Otorhinolaryngology, Juntendo University Faculty of Medicine, 2-1-1 Hongo, Bunkyo-ku, Tokyo 113-8421, Japan

**Keywords:** Carvenous sinus thrombosis, Sphenoiditis, Contralaterally, Endoscopy sinus surgery

## Abstract

**Objective:**

To report a rare case of unilateral cavernous sinus thrombosis caused by contralateral sphenoid sinusitis.

**Case report:**

A 33-year-old female visited our hospital for severe, right-sided, temporal headache, chemosis, periorbital edema, and proptosis. These signs were associated with congested erythematous nasal mucosa with purulent discharge from the right superior nasal meatus. Contrast enhanced CT showed dilated left superior ophthalmic vein, suggestive of thrombosis, contrast enhancement of the left cavernous sinuses, and dilation of cavernous sinus, indicating cavernous sinus inflammation. The right maxillary, ethmoid and sphenoid sinuses showed mucosal thickening and retention of purulent material. She was diagnosed with cavernous sinus thrombosis caused by contralateral sphenoid sinusitis. All clinical symptoms and signs improved after endoscopic sphenoidotomy and appropriate medical treatment.

**Conclusions:**

Sphenoiditis can cause contralateral cavernous sinus thrombosis. Early surgical sphenoidotomy and aggressive medical treatment are the cornerstones of successful management of this life-threatening complication.

## Background

Cavernous sinus thrombosis (CST) is a rare infective disease [1]. Although it was associated with high mortality and morbidity rates in the pre-antibiotic era, these rates have remained high in the modern era [2]. Thus, early diagnosis of CST is important [3]. The clinical findings of sudden proptosis, accompanied by erythema of the eyelid, chemosis, history of proptosis, and restricted ocular movement should alert the clinician to the possibility of CST. Imaging studies, such as contrast enhanced computed tomography (CT) and magnetic resonance imaging (MRI) have had a significant impact on the diagnosis of CST in recent decades [1]. CST can result from infection of any of the tissues drained by the cavernous sinus. This includes the mid-face, orbit and sinonasal cavity [2]. Early reports showed that only 15 percent of CST originated from the paranasal sinuses, while most cases were secondary to nasal or mid-facial skin infections. However, more recent reports suggest that sinusitis is currently the most common etiological factor in CST, possibly as a result of marked reduction in complicated facial skin infections associated with the use of antibiotics [4,5]. When the paranasal sinuses are the cause of CST, the ethmoid and sphenoid sinuses are often responsible. The mechanism of spread is either through direct extension or by retrograde thrombophlebitis along the ophthalmic veins.

Sphenoid sinusitis occurs in a significant proportion of cases of CST, either on its own or in conjunction with other sinus involvement. We experienced a case of CST caused by contralateral sphenoiditis. This case is quite rare, based on the literature review.

### Case report

**Figure 1:**
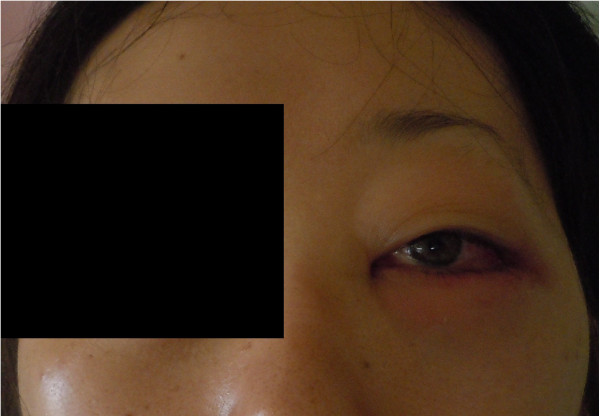
**Left eye lid at initial presentation.** At initial presentation, the left eye showed edema of the upper and lower lids as well as periorbital edema, chemosis and congestion of the conjunctiva.

A 33-year-old female visited our hospital for severe, right-sided, temporal headache, chemosis and proptosis in the left eye. Physical examination showed body temperature of 40.5°C, periorbital edema and chemosis in the left eye, left abducent nerve paresis, both pupils were equal, round and reactive to light and accommodation (Figure 1). Congestion of the erythematous nasal mucosa, and purulent nasal discharge drained from the right, contralateral, superior nasal meatus. Cranial nerve examination showed no paralysis and ophthalmological examination showed no abnormalities. Laboratory tests showed leukocyte count of 21700/mm^3^, C-reactive protein 15.0 mg/dL, and d-dimer, 54.8 mg/dL. No other disease was evident clinically.

**Figure 2:**
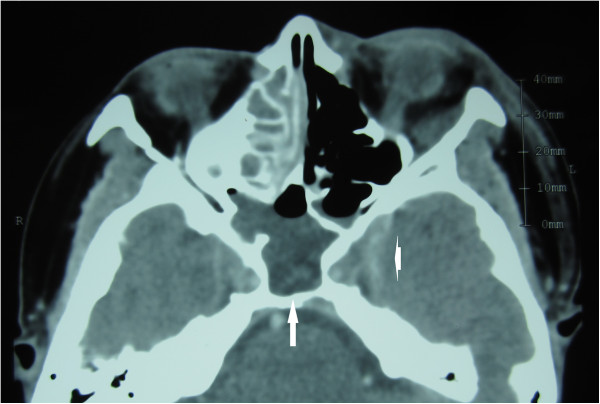
**Contrast-enhanced CT.** Contrast-enhanced CT showed enhancement and dilation of left cavernous sinus (arrowhead). Sphenoid sinus occupied the low density area. The right sphenoid sinus extended posteriorly to the left sinus and was in contact with both cavernous sinuses (arrow).

**Figure 3:**
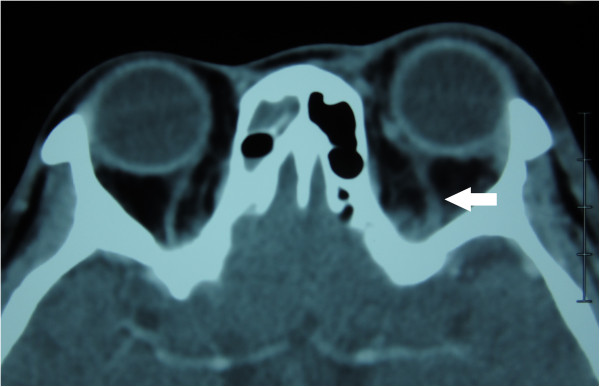
**Contrast-enhanced CT.** CT shows dilation of the left superior ophthalmic vein (arrow).

**Figure 4:**
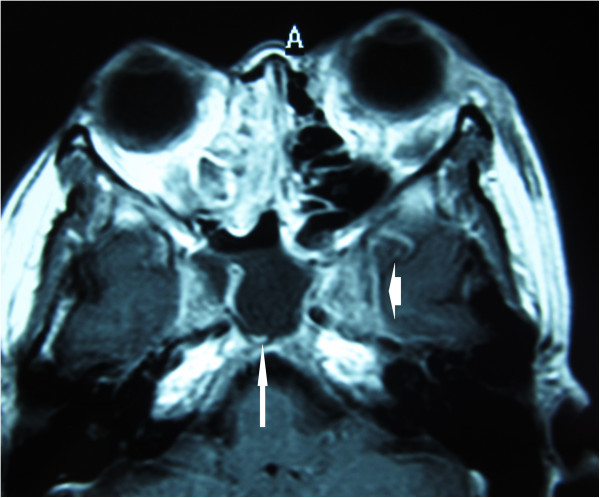
**Contrast-enhanced MRI.** Contrast-enhanced T1 MRI showed enhancement of the left cavernous sinus with some defects.

**Figure 5:**
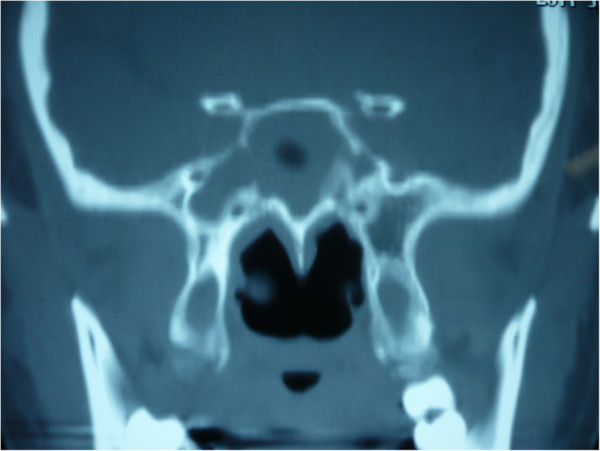
**Pre-operative coronal CT in bone window.** Coronal CT showed the right sphenoid sinus was dominant and much larger than the left.

Contrast enhanced CT of the orbits showed right proptosis and fat stranding in the left orbit. The left superior ophthalmic vein was enlarged, suggesting thrombosis. Contrast enhancement was noted within left cavernous sinuses and dilation of the cavernous sinus, indicative of inflammation of the cavernous sinus. Paranasal sinuses were clear on the left side. In contrast, the maxillary, ethmoid and sphenoid sinuses on right side showed mucosal thickening and retention of purulent material. The right sphenoid sinus was predominant and occupied the area underneath the cavernous sinus. Magnetic resonance imaging (MRI) confirmed the CT findings. Post-contrast T1 images showed enlargement of the left cavernous sinus with some defects, representing thrombosis of the left cavernous sinus. Coronal CT showed the right sphenoid sinus was dominant and much larger than the left (Figures 2, 3, 4 and 5).

**Figure 6:**
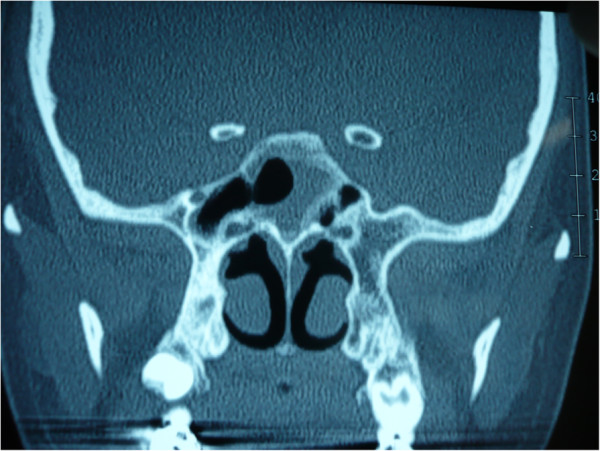
**Post-operative coronal CT in bone window.** In post-operative CT displayed the right sphenoid sinus was drained by operation.

The patient was immediately treated with intravenous ceftriaxone sodium, betamethasone and a therapeutic dose of low-molecular weight heparin. On the next day, bilateral endoscopic sinonasal surgery under general anesthesia was performed. Intraoperatively, the left sphenoid sinus and ethmoid sinus were checked, any accumulation of pus and mucous thickening by inflammation were not seen in both sinuses, whereas the right sphenoid and ethmoid sinuses contained pus and were lined with polypoid mucosa. The right sphenoid and ethmoid sinuses were opened widely and mucopurulent discharge was drained to clean the sinus. No surgical procedure was given for bilateral maxillary sinuses. After nasal packing, operation was finished (Figure 6).

Repeated cultures of samples obtained from the sphenoid sinus for microorganisms identified the presence of methicillin-sensitive *Staphylococcus aureus*. However, due to the seriousness of the condition, the patients received a 14-day course of pazufloxacin mesilate.

Endoscopic drainage of the sphenoid sinus resulted in marked improvement in the clinical symptoms and signs. The low-molecular-weight heparin was continued for 10 days post-operatively, but was later replaced with warfarin, which was continued for another 3 months, with the aim of achieving the target international normalized ratio of 2–3. Inflammation-related blood test improved after surgery, reaching normal ranges at 7 days after surgery. The patient was discharged from hospital on 12 post-operative day and was examined regularly during the follow-up period in the Outpatient Department. One month later, MRI showed no evidence of thrombosis of the cavernous sinus. One year later, the patient was in good condition without any morbidity.

## Discussion

CST is a relatively rare but life-threatening cause of cavernous sinus syndrome [6]. The mortality rate remains high, and significant morbidity includes residual cranial nerve palsies and blindness. Thus, early diagnosis and medical intervention are crucial.

The primary source of sepsis may be a distant focus or neighboring region [7]. CST may result from any infection of the tissue drained by the cavernous sinus. This includes the face, tonsils, soft palate, teeth, and ears. Once antibiotic therapy became widely available, however, the sphenoid sinus emerged as the most common primary source of infection predisposing to CST [7]. The sphenoid sinus has important anatomical relationships with the cavernous sinus. As a midline structure, it can present with bilateral or contralateral intracranial complications. Isolated sphenoid sinusitis is rare, nevertheless, sphenoid sinusitis occurs in a significant proportion of cases of septic CST, either in isolation or in combination with involvement of other sinuses [4,8,9]. The two cavernous sinuses are formed by the separation of the layers of the dura mater. In some areas, the cavernous sinus may be separated from the sphenoid air sinuses by only small amounts of soft tissue [8]. Sphenoid sinusitis can cause CST through a combination of factors. Spread of infection from the sphenoid sinus proceeds mainly through the afferent veins to the unvalved cavernous sinus and results in a classic fulminant CST. Microorganisms can spread directly from an infected sphenoid sinus to the cavernous sinus via communicating veins, via osteomyelitis of the intervening diploic bone, or by breaching mucosa in the presence of bony defects [10]. Sphenoid sinusitis produces very few localizing symptoms or external signs. Thus, sphenoid sinusitis is notoriously difficult to diagnose by routine clinical and radiological examination. Consequently, sphenoid sinusitis is frequently misdiagnosed on presentation; patients are referred initially to clinicians other than otolaryngologists, and the condition is suspected only after the development of complications. The resultant delays in instituting appropriate medical and surgical treatments for sphenoid sinusitis may explain why CST caused by this primary infection appears to have a poorer prognosis than CST caused by other etiologies [4,5,8,9]. In our case, intra-operative remarks showed that no abnormality was seen in the left sphenoid sinus and the right sphenoid sinus contained pus and was lined with polypoid mucosa. CT showed that the right sphenoid sinus was the dominant sinus, extending posterior to the left sphenoid sinus and lying in contact with both cavernous sinuses. This could perhaps explain the reason for the spread of infection to the left cavernous sinus.

The diagnosis of CST is best established on clinical grounds and confirmed by appropriate radiographic studies. High-resolution contrast-enhanced CT or MRI is useful in the assessment of cases with clinical features of cavernous sinus thrombosis. Contrast-enhanced CT scan may reveal the primary source of infection, thickening of the superior ophthalmic vein, and irregular filling defects in the cavernous sinus [11,12]. However, MRI is more sensitive than CT in the detection of septic CST because it can demonstrate details of the blood vessels and provide more multiplanar sections. Direct signs of CST on MRI include changes in signal intensity and in the size and contour of the cavernous sinuses; while indirect signs include dilatation of the tributary veins, exophthalmos, and increased dural enhancement along the lateral border of the cavernous sinuses [12,13]. However, the CT and MRI findings may be normal, especially early in the disease course. The imaging evidence of bilateral CST is shown, even when clinical manifestations are limited to one side. In our patient, radiographic examination did not clearly show thrombosis of the cavernous sinus. However, inflammation of the cavernous sinus and dilation of the superior ophthalmic vein were noted by CT and MRI. Sphenoid sinusitis was observed contralaterally. Sphenoid sinusitis was regarded as the cause of CST, due to the predominance of the right sphenoid sinus and its location beneath the cavernous sinus.

The consensus on treatment of septic cavernous sinus thrombosis is that it should include high-dose intravenous antibiotics directed at the most common pathogens associated with the inflammation and surgical drainage of the source of infection in the paranasal sinuses [6]. Appropriate selection of empirical antimicrobial therapy should also take into account the source of primary infection. Antibiotics should be administered for an extended period, at least two weeks beyond the time of clinical resolution, because bacteria sequestered within the thrombus may not be killed until the dural sinuses have started to recanalize [14]. Surgery is indicated for drainage of the primary site of infection. Surgical intrusion into the cavernous sinus is difficult and not recommended. If sphenoid or ethmoid sinusitis is documented on CT scans, surgical drainage of these infected pockets should be performed promptly. Endonasal sinus surgery (ESS) is an essential step in the treatment of these patients. The procedure restores the sinuses to their normal physiologic state by providing adequate aeration and restoration of the normal mucociliary flow [15]. ESS adequately drains the infected sinuses with little morbidity. If the patient cannot withstand general anesthesia, ESS can be performed under local anesthesia. In our case, rapid improvement occurred after ESS and intravenous antibiotics improved the clinical condition without any morbidity. We consider the combination of antibiotics and ESS optimal treatment strategy for CST caused by paranasal sinusitis. However, heparin was administered to the patient, although the use of anticoagulants remains controversial.

## Conclusions

The diagnosis of CST remains difficult despite available imaging techniques. Sphenoid sinusitis is an uncommon etiology and easily missed on initial evaluation and CST can occur by spread of contralateral sphenoid sinusitis. With regard to suspicion of CST based on clinical findings, otolaryngologist should regard not only ipsilateral infection but also contralateral paranasal sinus infection. Once CST is diagnosed, early and aggressive intervention is crucial; including the use of intravenous antibiotic therapy combined with ESS.

## Consent

Written informed consent was obtained from the patient for publication of this report and any accompanying images.

## Competing interests

The authors declare that they have no competing interests.

## Authors’ contributions

HK: performed surgery. documented the case. drafted the manuscript. FM: surgery performed and documented the case. Drafted the manuscript. KK: exams performed the analysis of the image. MK: exams performed the analysis of the image. DS: review of literature. drafted the manuscript. KI: conducted a review of literature. drafted the manuscript. All authors read and approved the final manuscript.
